# Current Challenges of Vaccination in Fish Health Management

**DOI:** 10.3390/ani14182692

**Published:** 2024-09-16

**Authors:** Avnish Kumar, Sushil Kumar Middha, Soumya Vettiyatil Menon, Biswaranjan Paital, Shyam Gokarn, Meghana Nelli, Rakshith Bangalore Rajanikanth, Harish Mani Chandra, Susithra Priyadarshni Mugunthan, Sanwar Mal Kantwa, Talambedu Usha, Akshaya Kumar Hati, Divyadharshini Venkatesan, Abira Rajendran, Tapas Ranjan Behera, Swarupa Venkatesamurthy, Dipak Kumar Sahoo

**Affiliations:** 1 Department of Biotechnology, School of Life Sciences, Dr. Bhimrao Ambedkar University, Agra 282004, India; 2 Department of Biotechnology, Maharani Lakshmi Ammanni College for Women, 18th Cross, Malleswaram, Bangalore 560012, India; 3 Department of Chemistry and Biochemistry, School of Sciences, Jain University, #34 JC Road, Bangalore 560027, India; 4 Redox Regulation Laboratory, Department of Zoology, College of Basic Science and Humanities, Odisha University of Agriculture and Technology, Bhubaneswar 751003, India; 5 Department of Biotechnology, Thiruvalluvar University, Serkkadu, Vellore 632115, India; 6 Department of Zoology, B. S. Memorial P.G. College, NH 52, Ranoli, Sikar 332403, India; 7 Department of Biochemistry, Maharani Lakshmi Ammanni College for Women, 18th Cross, Malleswaram, Bangalore 560012, India; 8 Dr. Abhin Chandra Homoeopathic Medical College and Hospital, Homeopathic College Rd., Unit 3, Kharvela Nagar, Bhubaneswar 751001, India; 9 Department of Community Medicine, Fakir Mohan Medical College and Hospital, Januganj Rd., Kalidaspur, Balia, Balasore 756019, India; 10 Department of Veterinary Clinical Sciences, College of Veterinary Medicine, Iowa State University, Ames, IA 50011, USA; dsahoo@iastate.edu

**Keywords:** fish vaccine, aquaculture, antibiotic, fish infectious diseases, environmental safety, global market

## Abstract

**Simple Summary:**

About a 4% decline in fish production was recorded in 2020 in comparison to 2018 in inland aquaculture sectors. In 2014, about 10% of aquatic cultured animals were lost (costing global annual losses > USD 10 billion) due to infectious diseases. Contagious diseases generate several stresses, including oxidative stress in fishes that hampers their reproduction, growth, and production value. Aquaculture management needs to employ preventive measures against various contagious diseases for maintenance. Therefore, vaccination in fish, especially in broodstocks, is one of the most important measures to curb such fish losses. Here, we reviewed the current status of fish vaccines and the modern technology used to produce (alternative) fish vaccines, such as plant-derived vaccines for fish health management. Reliable aquaculture vaccines to increase economic productivity in aquaculture sectors are needed. Emerging contagious diseases in different fish species demand novel vaccines to be produced by adapting biotechnological approaches. Vaccination in broodstocks is important, and the fish vaccines are produced with whole-killed pathogens; subunits of a protein, peptide, or a recombinant protein; DNA vaccines; or live attenuated vaccines via different modes of administration. The use of adjuvants in fish vaccines needs to be researched.

**Abstract:**

Vaccination is an essential method of immunological preventive care required for the health management of all animals, including fish. More particularly, immunization is necessary for in-land aquaculture to manage diseases in fish broodstocks and healthy seed production. According to the latest statistics in 2020, 90.3 million tons of capture fishery production was achieved from the aquaculture sector. Out of the above, 78.8 million tons were from marine water aquaculture sectors, and 11.5 million tons were from inland water aquaculture sectors. About a 4% decline in fish production was achieved in 2020 in comparison to 2018 from inland aquaculture sectors. On the other hand, the digestive protein content, healthy fats, and nutritional values of fish products are comparatively more affordable than in other meat sources. In 2014, about 10% of aquatic cultured animals were lost (costing global annual losses > USD 10 billion) due to infectious diseases. Therefore, vaccination in fish, especially in broodstocks, is one of the essential approaches to stop such losses in the aquaculture sector. Fish vaccines consist of whole-killed pathogens, protein subunits, recombinant proteins, DNA, or live-attenuated vaccines. Challenges persist in the adaption of vaccination in the aquaculture sector, the route of administration, the use of effective adjuvants, and, most importantly, the lack of effective results. The use of autogenous vaccines; vaccination via intramuscular, intraperitoneal, or oral routes; and, most importantly, adding vaccines in feed using top dressing methods or as a constituent in fish feed are now emerging. These methods will lower the risk of using antibiotics in cultured water by reducing environmental contamination.

## 1. Introduction

**Figure 1:**
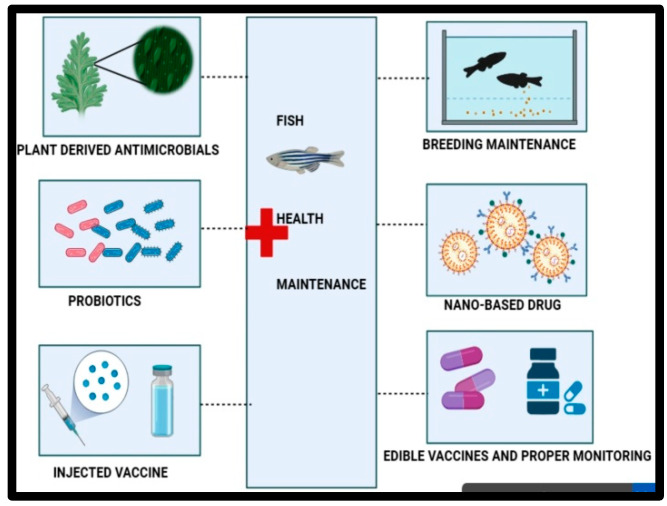
Management of fish health with multiple approaches. This panel depicts ecosystem-level management, which includes disease and environmental control measures.

The rate of infectious diseases is higher in animals, which is very dangerous, as they may affect humans [1,2,3,4,5]. Some infectious agents and their causative diseases are explained in Table 1. To develop immunity in animals (including fish) and reduce the susceptibility of animals to communicable diseases, vaccination is crucial. Thus, vaccines have a significant role in managing live stocks (Figure 1). Vaccines have been applied as preventive measures to reduce the chance of diseases such as rabies and rinderpest, as well as other zoonotic ones that can be endemic [2,3,4,5,6,7,8,9,10,11,12,13,14].

**Figure 2:**
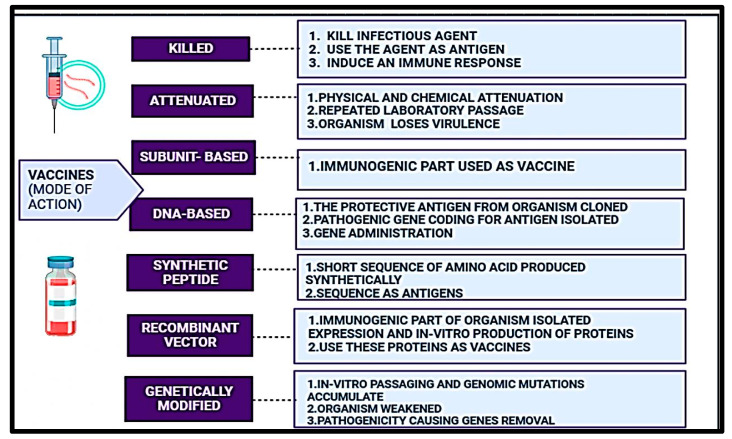
Vaccine classification based on their mode of action and their sources. This Figure is modified after Assefa and Abunna [21].

Even though vaccines are available for animals based on the type of disease, prioritizing them is necessary. Irrespective of fish, seaweed, and crustaceans being vital food sources that provide protein, the aquaculture industry suffers from bacterial diseases, viruses, parasites, and fungi. Fish vaccines are limited in quantity and are rendered inactive by factors such as temperature, pH, and organic waste. Despite thorough investigation, only a limited number of authorized vaccines and antivirals demonstrate efficacy against viral infections [15]. Research on fish immunity mainly relies on vaccination, gene/protein sequencing, antigen screening, vaccine administration, and enhanced aquatic immunizations [16,17]. Numerous vaccines have been developed, such as live vaccines and recombinant vaccines, to prevent these infectious diseases, but still, the recovery rate from contracting the diseases is meager [15,18]. For example, commercial vaccines LF-89 and EM-90 of *P. salmonis* may not show protective efficacy [19]. TiLV (tilapia lake virus) vaccination can cause the virus to return to a virulent condition, retain some virulence, or cause virulence in fish with weakened immune systems [20,21]. Nevertheless, a wide range of commercial vaccines are available according to animal infectious disease types and infection rates (as given in Table 1). Still, the availability of such vaccines in fish may be able to curb the contagious diseases that affect their production and reproduction value (Figure 2).

Total fish weight is estimated to double in India, Mexico, and Brazil by 2050, as stated by the FAO’s food balance sheets, and the problems of total fish demand for the top 10 countries ranked in order of human consumption is expected to be solved by that time [21,22,23,24,25,26]. The controlled production of aquatic organisms such as fish, algae, mollusks, and other aquatic plants is known as aquaculture or aquafarming. In contrast to commercial fishing, which includes wild fish capturing, aquaculture involves raising populations of freshwater, brackish water, and saltwater species in controlled or semi-natural environments. However, their production is hampered severely by several (infectious) diseases.

Aquaculture ecosystems can naturally be home to many diseases, which have adverse economic effects on productivity. Diseases are also can be contagious in oceanic ecosystems. In hosts, different levels/percentages of infections occur; for example, 49% of fish, 21% of crustaceans, 28% of mollusks, and 1% of echinoderms are susceptible to various diseases [27,28]. Exotic fish may become more susceptible to diseases in new environmental conditions than local wild varieties. Opportunistic infections may impair the aquaculture sector due to high stocking densities of monoculture systems, leading to greater host contact with pathogenic organisms, which in turn cause increased stress for the fish and degrade the water quality [27,28]. The main objective is to prevent the occurrence of diseases in aquaculture, and this objective can be met by maintaining good water quality, moderating stocking density, producing disease-resistant, genetically modified stocks, using immuno-stimulants and therapeutics against fish pathogens, and utilizing vaccines against bacteria, viruses, and other pathogens in fish [27,28].

A vaccine can be a suspension of weakened or killed microorganisms, toxins, or any other biological preparation that consists of a protein or a polysaccharide antigen to induce a protective immunological response against the infecting pathogen [29]. Currently, three routes of administration of vaccines to fish, such as oral vaccines, are incorporated into the feed. Immersion vaccines are applied by dipping the fish for a short time in a concentrated solution of vaccine, and injection vaccines, as the name suggests, are injected into the fish. This current review attempts to update information on fish vaccines and their efficacy and prospects using a systematic literature survey [27,28,29]. It is to be noted that different commercial veterinary vaccines available at present, such as canine (Canileish Leishmania), avian (Trovac Flu), equine (Proteq Flu), salmonoid (Apex IHN), food (iPED+), and feline (PureVax rabies) vaccines, are available currently [30].

## 2. Literature Review Process

This study systematically reviews the literature on fish vaccines, utilizing a comprehensive search conducted across PubMed, AGRICOLA, Google Scholar, Scopus, Science Direct, and Web of Science. Search terms included “vaccines”, “fish vaccines”, and “fish”. The review encompassed both peer-reviewed and respected non-peer-reviewed English-language sources. The review examined English-language peer-reviewed publications, books, journals, and credible websites. Due to their enormous biomedical and scholarly collections, PubMed/MEDLINE and Google Scholar were used as primary sources. AGRICOLA, with 6 million records in agricultural and biological sciences, was especially important. The search technique adhered to current review procedures and included extra phrases such as “disease”, “immunity”, and “economic loss”. From AGRICOLA, 10,000 publications on “fish”, 1553 on “vaccine”, and 26 on “fish vaccines” were examined. PubMed returned 4018 hits, whereas Google Scholar returned nearly 489,000, all of which were filtered for relevance. The data were divided into subsections covering many areas of fish vaccines, such as the condition of aquaculture, present fish illnesses, and future possibilities [25,31,32]. Of the first findings, 187 articles were selected based on their direct relevance to fish vaccinations. The evaluation process examined the origins and development of animal and fish vaccines and their efficacy, commercialization, and influence on fish immunity, illness prevention, and economic value.

These publications were selected, categorized, and analyzed to offer a thorough overview, emphasizing the importance of fish vaccines and their future potential in aquaculture.

## 3. Need for Fish Vaccines

### 3.1. Status of Aquaculture Products

Fish are essential for ecosystem balance, economic contribution, water pollution prevention, disease control, and food sources [27]. Fish are also an essential source of byproducts like fish oil, liver oil, fish protein, fish flour, fish roes, fish silage, fish fertilizers, isinglass, etc. However, they face threats like climate change, habitat loss, overfishing, illegal fishing [33], ocean pollution, and disease. It is essential to protect them from these threats, and, in this concern, vaccination strategies must be developed to safeguard their existence.

According to the FAO, [25] world fisheries and aquaculture production increased from 110.7 metric tons to 178.9 metric tons in the years from 1990 to 2018 but slightly decreased the production of aquaculture and fisheries in the year 2019 (1.5 metric tons) and to 1.1 metric tons in 2020. Human consumption of aquaculture and fishery products increased from 86.6 metric tons to 158.1 metric tons from 1990 to 2019 but reduced in 2020 to 0.7 metric tons. The trade (export) of fisheries and aquaculture also increased from 1990 to 2018, from 39.6 metric tons to 66.8 metric tons, but decreased to 0.2 metric tons in 2019 and to 6.8 metric tons in 2020. The unusual COVID-19 outbreak caused lockdowns and market, port, and border closures worldwide. Commerce plummeted, disrupting aquatic food production and distribution and causing unemployment and livelihood losses. Furthermore, the “UN Decade of Ocean Science for Sustainable Development (2021–2030)” identifies three key challenges to reduce global biodiversity losses: involving diverse stakeholders, ensuring adequate resources, and developing a transparent monitoring system for progress [25,33].

India is the second largest fish-producing country after China. China produces 58.8 million metric tons (one-third of total world fish production), while India produces 9.46 million metric tons annually, although the aquaculture industry was established in the 19th century in India [34]. According to the Ministry of Fisheries, Animal Husbandry and Dairy, Government of India, fish production increased from 6,305,000 metric tons to 14,164,000 metric tons in just 16 years, i.e., from the year 2004 to 2020 [34].

Contagious and infectious diseases are still a significant threat to the development of the aquaculture industry in the world. Fish are vulnerable to many bacterial, viral, fungal, protozoan, and parasitic diseases like red pest, fungus, tuberculosis, argulus, velvet or rust, anchor worm, costia (*Ichthyobodo*), lymphocytes, etc. Some communicable diseases, along with their associated pathogens, symptoms, and treatments, are given in Table 2.

### 3.2. An Emergency Need for Fish Vaccines

Aquatic foods like sea plants, fish, and crustaceans are one of the largest sources of protein for humans. However, the aquaculture industry faces substantial economic losses due to infectious diseases. In aquaculture, bacteria account for more than 50% of infectious diseases, followed by viruses, parasites, and fungi [51]. These diseases impact fish internally and topically, including their gills, fins, and body surfaces. In general, fish farmers have used a variety of strategies such as biosecurity controls, better settings, and feeding the fish special diets to manage the diseases that affect fish. The use of disinfectants, the addition of antibiotics, and treatment with pharmaceuticals like therapeutic treatments are a few examples of synthetic methods [25]. Although antibiotics and chemotherapeutics have been used to treat diseases, there are some downsides, such as problems with drug resistance and consumer safety concerns [52,53,54,55]. Successful vaccination delivery and design are crucial, and these depend on aquatic species’ immunological responses, as well as proper dosing. Aquaculture vaccine research and deployment require particular vaccine-delivery methods with regard to vaccine technology, aquatic organism species and their reproductive stages, pathogen nature, infection pathways, and cost [56]. Nevertheless, a few fish vaccines are being studied and are available in the literature (Table 3).

In Table 3, we can see that the majority of vaccines are produced by inactivating bacterial and viral cultures, which are injected intra-peritoneally for emulsion-based injectable vaccines and intramuscularly for DNA plasmids [62], which produce higher protection rates against diseases for fish. However, using an injection to administer the vaccines in fish, even fingerlings, is tricky. Even with the automation of the vaccination process in fish, they still experience stress. However, economically important fish can be vaccinated [63]. Environmental conditions such as pH, the presence of organic matter, and temperature can impact a drug’s efficacy [64,65]. Viral infections are more challenging to control due to the lack of antivirals or efficient viral vaccinations [66]. Only a few viral vaccines have acquired licenses, even though companies and academic institutions have carried out numerous research investigations to create viable viral vaccines [67].

Vaccination is an effective method to improve the immune response and is cost-effective for aquaculture, but it should be developed to overcome the hurdle mentioned earlier. Aquatic vaccines will likely foresee advancement in the following years because of increased technological inventions like sequencing, antigen screening, the development of fish cell lines, the development of innovative vaccine protein expression and delivery systems, and increased fundamental knowledge about fish mucosal immunity.

## 4. Developments of Commercially Available Vaccines and Experimental Available Vaccines

A shortage of anti-viral medicines, efficient viral vaccinations, and illness resistance makes viral infections harder to treat. Unlike bacterial infections, viral illnesses in animals or fish have less effective treatment systems [60]. Therefore, the vaccination of fish, particularly broodstocks, is one of the most important ways to reduce losses in the aquaculture business. This is because the broodstocks of aquatic animals are used to produce and supply seeds from that species for most aquaculture systems [68]. Therefore, healthy and immunized seeds can be obtained from such immunized broodstocks.

This section provides current research on protein-based, live-attenuated, virus-like particles, bacterins, DNA fish vaccines, etc. These topics cover present knowledge of fish vaccine administration methodologies, effective adjuvants, and obstacles in adapting vaccines for the fishing industry, including studies on mucosal immunity. Novel techniques encompass intramuscular, intraperitoneal, oral, and feed-based autogenous vaccines. Furthermore, bioinformatics and in silico testing have accelerated the vaccine development process. Vaccination practices have been increasing in the aquaculture business.

Due to various legal guidelines, several farms have been routinely vaccinating their fish, which, when administered appropriately, provides a high level of protection. Approximately 30 vaccines were reported to offer protection for fish against significant bacterial and viral illnesses, meeting the need for continuous aquaculture despite the presence of disease-causing agents and environmental pressures [69]. With regard to enteric red mouth and vibriosis, the concept of immunizing fish on a commercial scale has finally been realized (Table 1, Figure 1 and Figure 2).

### 4.1. Early Works on Fish Vaccination

Fish were immunized for the first time successfully via an oral vaccine in carp and trout against *Aeromonas punctata* in 1938 and *Aeromonas salmonicida* in 1942, respectively [70]. The vaccinations against enteric red mouth disease (ERM), also known as yersiniosis and vibriosis (and sometimes only referred to as yersiniosis), were the first bacterial vaccines to be made available for commercial purchase in the United States in the late 1970s [71,72]. Bioveta, a Czechoslovak business, created the first viral immunization for fish in 1982 [70,71,72,73]. The vaccine was created by the fusion of two deactivated spring viremia of carp SVC virus strains in oil, followed by injection. The vaccination included no live virus. The only carp vaccine currently in the market for commercial sale in Asia is an inactivated injection that protects against grass carp bleeding disease caused by the reovirus. The manufacturer of a vaccine for koi herpesvirus for purchase in Israel uses an attenuated strain of CyHV-3 [70]. The history, types, and modes of administration of vaccines in fish are different for different vaccines [72]. Key researchers in this area have identified the application of adjuvants and immunostimulants in fish vaccines and their delivery methods. The data were obtained based on alternative methods (other than injection) for vaccine delivery and the protective efficacies of traditional and promising new-generation adjuvants [74,75].

Infectious illnesses have a negative effect on aquaculture practices all around the world. Pathogenic illnesses in aquaculture in India lead to considerable financial challenges and threaten the survival of fish populations. Antibiotic resistance patterns, residual effects, and environmental impact have all increased the need for establishing appropriate alternate preventive methods [76,77,78,79,80,81,82,83,84]. Vaccinations have been shown to be an effective technique for disease control, allowing for increased fish productivity. *Aeromonas hydrophila* is a widely spread pathogen in all farmed fish species in India. Most vaccine research has focused on bacterial illnesses, including *Aeromoniasis* and *Edwardsiellosis*. Despite significant efforts and potential pilot programs for developing vaccines against several parasitic and fungal infections, India’s aquaculture sector has no universally applicable immunizations. Indian researchers are attempting to develop a vaccine to protect the fish business, listing the problems associated with manufacturing and exploring the opportunities of earning money from such vaccines [53]. Tafalla et al. [75] defined and listed the evolution of commercially available fish. More than 26 licensed fish vaccines are available commercially and globally at present [17]. Vaccines have been approved for use by the United States Department of Agriculture (USDA) for diverse species of aquaculture, and most of them are produced by conventional methods that target pathogens via culture testing. This collection of vaccines has successfully protected fish against some severe fish diseases up to an extent.

### 4.2. Properties of Fish Vaccines and the Vaccination Process

Fish vaccinations provide protection against diseases and should be ecologically safe for both fish and their surrounding ecosystems, economically viable for large-scale production, easy to administer, and capable of eliciting a robust immune response in delicate species, with few adverse effects, if any [70,71,72,73,74,75]. Molecular research demonstrated that immunoprophylactic approaches in grass carp stimulate viral nucleic acid sensors, TLRs, HMGBs, RLRs, and PRRs, enhancing virus resistance [84]. Significantly, these approaches may be improved to provide the highest level of security. Toll-like receptors (TLRs) detect pathogen-associated molecular patterns (PAMPs) and initiate immunological signaling cascades that enhance innate immunity and adaptability [70,71,72,73,74,75]. TLR adjuvants and activators can enhance the immunization of fish and other aquatic animals [70].

### 4.3. Types of Fish Vaccines

Fish vaccines can be protein, live-attenuated vaccines, virus-like particles, bacterines, and DNA vaccines. Challenges remain in the fishing industry regarding the adaptation of vaccination, including the method of administration, the use of effective adjuvants, and, most importantly, the lack of data from more focused research on the mucosal immunity mechanism [15,85]. The use of autogenous vaccinations, which include intramuscular, intraperitoneal, and oral vaccinations and, most significantly, the incorporation of vaccines in feed via top dressing methods in feed or as a constituent in fish feed, are all examples of emerging practices [70,75].

#### 4.3.1. Whole-Cell Vaccines

Whole-cell vaccines that have been killed or inactivated can be characterized as a suspension of pathogens that have been destroyed (either by heat or chemical treatment) but still provide protection when administered to the host. These vaccines are cheap to make. Pathogens such as *Vibrio anguillarum*, *Vibrio salmonicida*, *Vibrio ordalli*, *Yersinia ruckeri*, and *Aeromonas salmonicida* are combated by applying these vaccines to fish [86,87] (Table 4). These killed vaccinations are commercially available as formalin-inactivated whole-cell vaccines, which can be given with or without adjuvants. Vaccines against deadly fish viruses, including infectious pancreatic necrosis virus (IPNV), infectious hematopoietic necrosis virus (IHNV), viral hemorrhagic septicemia virus (VHSV), and spring viremia of carp virus (SVCV) have been produced. When the inactivated IPNV was injected into rainbow trout fry, the fish were well protected [88], and when it was given to brook trout with Freund’s complete adjuvant, the fish obtained a robust humoral response, but this did not stop them from spreading the virus [89,90]. Formaldehyde is used to sterilize fish. Although vaccination with inactivated vaccines effectively prevents the spread of diseases, this method is deemed unsuitable for some viruses because of the age at which symptoms first appear. The humoral immune response is induced by killed vaccines and, to maintain protection, booster dosages are required. The primary drawbacks of heat-killed vaccines are the high cost of cell culture manufacture, difficulty of purification, and limited administration options (Table 4).

#### 4.3.2. Attenuated Vaccines

Attenuated vaccines are genetically or chemically weakened microbes that can produce short-lasting immune responses in the host [52]. They contain live bacteria and viruses that no longer cause illness.

#### 4.3.3. Recombinant Vaccines

Recombinant vaccines are those that use only the immunogenic areas of the pathogen to be produced in a heterologous host and are then utilized as vaccines [17]. Additionally, recombinant vaccines can be genetically modified organisms (bacteria or virus) with reduced pathogenicity, thus being recombinant and attenuated to produce vaccines.

#### 4.3.4. Synthetic Peptide Vaccines

Synthetic peptide vaccines can act as subunit vaccines, antigenic sites, or peptides that have been tested to induce antibodies against infectious pathogens such as nodavirus, rhabdovirus, birnavirus, IHNV, IPNV, and VHS [17].

Among various vaccines for fish, mRNA vaccines are in colossal demand compared to conventional vaccines. Live-attenuated vaccines (LAVs) successfully reduce diseases, but their safety and efficacy are still doubtful. The mRNA vaccine, on the other hand, is non-infectious; hence, it is safer.

#### 4.3.5. DNA Vaccines

DNA vaccines containing plasmids that carry a pathogen’s antigen have garnered attention as a potential way to boost fish defense against illnesses [89]. Aquaculture researchers have boosted VHSV and IHNV resistance by injecting viral genes expressing surface glycoproteins intramuscularly (IM). VHSV glycoprotein and DNA immunization elicited an immune response in rainbow trout [17].

#### 4.3.6. Mucosal Vaccinations

Mucosal vaccines against pathogenic infections are being developed because they can elicit protective responses at mucosal surfaces by preventing pathogen reproduction [90]. Oral, immersion, and nasal teleost fish mucosal vaccines stimulate B and T cells to induce systemic and mucosal responses. It has been shown that mucosal vaccines work if they imitate the natural route of infection, such as going through mucosal surfaces [74]. For example, rainbow trout (*Oncorhynchus mykiss*), a bony fish renowned for its specialized mucosal immune system, was studied by Magadan et al. [76]. The olfactory organ of this fish contains nasopharynx-associated lymphoid tissue (NALT), distinguished by a widespread network of myeloid and lymphoid cells. Myeloid and lymphoid cells activated by the nasal immunization showed distinctive dynamics of IgM and IgT repertoires at systemic and mucosal locales, demonstrating an unusual capacity to induce splenic Ig responses.

#### 4.3.7. Plant-Based Edible Vaccines

Plants might provide an inexpensive foundation for creating cost-effective, edible, live-attenuated, pathogen-free vaccinations that can reduce several pathogenic fish illnesses to maintain a sustainable aquaculture. Plant-based vaccines may minimize the frequency of live-attenuated virus or bacterial booster doses. Modern plant researchers suggest that plant cells could express mammalian proteins after cloning [77]. Specific plant cell cultures or whole plants can be used as expression hosts. To express mammalian genes in plants, cloned complementary DNA must be spliced with a plant promoter, terminator, or regulatory region. Selectable markers help to identify recombinants [78]. Plant-based vaccines would be easy and convenient to consume orally. Follicle-associated epithelium M cells will grab the protein unit to activate mucosal immunity [74,78].

#### 4.3.8. Nanoparticle-Based Vaccine

Nanoparticles provide tailored vaccination delivery, antigen stability, and adjuvant effectiveness. Vaccinating particular body parts with these systems might prevent disease transmission. Nanoparticles can increase weak antigen immunogenicity and release kinetics, stability, and targeted dispersion over traditional adjuvants. Oral immunization is preferred in aquaculture since each fish in a pond, especially small fish, cannot be injected with vaccines. Maintaining gastrointestinal stability is difficult with oral vaccines. Nanoparticles may solve this problem [79]. Alginate, chitosan, and PLGA have different chemicals mixed with antigens and immunostimulants to enhance delivery and immune responses.

### 4.4. Advancements in Vaccine Development

New biotechnology developments have enabled the quick production of vaccines against various pathogens. In bioinformatic approaches, software predicts the gene to produce recombinant proteins functional for vaccines [52]. Andreoni et al. [80] used this software-based approach to investigate vaccines against intracellular infections, such as *Flavobacterium columnare* and *Edwardsiella tarda* in *Photobacterium damselae* subsp. *piscicida* fishes. These cause the most common significant diseases of fish columnaris and Edwardsiellosis, respectively [90,91]. Both an in silico examination and a functional analysis could lead to the conclusion that an effective candidate gene exists, which could be prioritized to develop a vaccine to prevent a disease’s further spread. This would undoubtedly reduce the time required to develop a novel vaccine. There are approximately 30 vaccines available that have the potential to protect fish against the most lethal bacterial and viral diseases.

## 5. Contemporary Need for Vaccines in Fish, a Growing Field of Research

Aquaculture production was estimated to be 90.3 million tons worldwide, with a decline of 4% in 2020–2021 [25]. Etiological diseases in fish and their treatment methods are always challenging. The immunization of fish is necessary to combat typical diseases and maintain their renewable production for sustainability in aquaculture. The spreading of any infectious diseases in fish may be multiplied by environmental stress caused by pollutants. One of the species affected due to such stress is shrimp. A total loss of one billion US dollars was reported by Briggs et al. [91]. Infectious agents or parasites, such as bacteria, viruses, algae, etc., are generally present in water. Some of the primary etiological agents and infected species of fish are shown in Table 5 [92].

Reviews have focused on various vaccines for fish, among which mRNA vaccines are in huge demand compared to conventional vaccines [99,100]. Live-attenuated vaccines (LAVs), either commercially available or under experimental conditions, are noted to be successful in reducing diseases. Some examples are the Edwardsiella ictaluri vaccine (against enteric septicemia in catfish), arthrobacter vaccine (against bacterial kidney disease in salmonids), and *Flavobacterium columnare* vaccine (against columnaris infection in catfish). Nonetheless, the safety and efficacy of certain vaccines remain uncertain. The mRNA vaccine, on the other hand, is non-infectious; hence, it is safer [99,100]. Other types of vaccines used are nanoparticulate vaccines (nanogels, micelles) and liposomal vaccines (natural or synthetic lipids), which combat infectious diseases [101,102].

Some vaccines available for infectious diseases are inactivated (IPNV, ISAV), nucleic acid (DNA, RNA), and live-attenuated vaccines. DNA vaccines have been studied to be effective against viral infections and have been developed for a series of pathogens in water [103,104]. Even though numerous types of vaccines have been explored, tested, and controlled successfully for many diseases, enormous challenges persist in delivering effective immunization in fish. Disease outbreaks, mitigatory species of fish, anthropogenic activities, and morbidity rate increases are the challenges faced by farmed and traded aquatic species. Some activities, such as mining and construction works in nearby areas, lead to viral infections in marine ecosystems. Moreover, the consumption of infected fish, used as a protein source for farm fish, results in the infection of farm fish species. Another major drawback is the lack of disease diagnosis tools and surveillance measures, especially in underdeveloped countries [105,106,107]. Different management strategies and policies should be developed to manage aquaculture trading and diseases. A multifaceted approach must be designed to prevent further emerging etiological agents and the infectious diseases caused by them (Table 5).

## 6. Fish Vaccine Production against Various Pathogens

**Figure 3:**
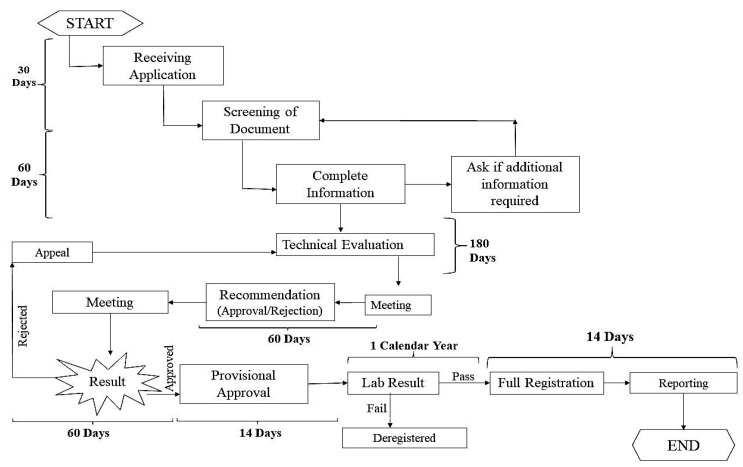
Schematic diagram for fish vaccine regulatory approval.

The discovery and subsequent production and commercialization of vaccines against infectious diseases in fish are commercially unavailable. These processes use multiple sources and steps. Each vaccine undergoes the steps of identification of the pathogen/disease, process development, production, validation with multiple individual fish, documentation, and vaccine licensing for commercialization (Figure 3). Various sources are used to achieve these steps.

### 6.1. Nocardiosis

Nocardiosis, caused by *Nocardia seriolae*, was first discovered in Japan’s central region [108]. It swiftly expanded to the country’s western areas, resulting in widespread Seriola fish mortality. Nocardiosis initially did not have a significant negative economic impact but is now considered a major cause of mortality in fish [109]. Although Seriola fish become easily infected by *Lactococcus garvieae*, nocardiosis has replaced it as the disease that causes the most economic harm to Japan’s aquaculture sector due to the development of a vaccine to control the bacteria [110]. Nocardiosis is distinguished by tubercles on the kidney and gills, as well as ulcers on the body’s exterior [111,112]. The creation of a vaccination against this illness is eagerly awaited. The administration of Freund’s incomplete adjuvant or formalin-killed cells of *N. seriolae* generated a humoral immune response, but the vaccine does not have a protective role against infectious diseases [113]. Numerous studies have documented that *N. soli*, *N. fluminea*, and *N. uniformis* could be vaccinated using live vaccines, such as in *N. seriolae*, a “codon-optimized antigen-85- like gene” [114]. Kato et al. [114] insisted on “DNA vaccines encoding the codon-optimized antigen-85-like” genotype of *N. seriolae* and malin-killed cell vaccine augmented with hybrid interleukin-12.

### 6.2. Bacterial Hemolytic Jaundice

It was documented that bacterial hemolytic jaundice in farmed yellowtails causes a 5–20% overall loss in cultured fish, like the Japanese amberjack population [115]. The hemolytic activity of the bacteria causes the skin and muscle tissue of the infected fish to turn yellow [116]. Similar infection may also occur in natural fish population [117]. The illness initially surfaced in the 1980s and has since spread rapidly over western Japan [16,118]. In addition to the losses caused by nocardiosis, a silent infection in yellowtail kingfish, the illness also results in considerable economic losses, as it affects a large number of fish before shipping. *I. seriolicida* exhibits low pathogenicity for greater amberjack and yellowtail kingfish [119].

Matsuyama et al. [117] reported that those fish that survive against naturally occurring contagions with *Ichthyobacterium* exhibit strong immunity and antibody-mediated death of the pathogen. Even though the bacterial serotype has not been identified, molecular epidemiological studies have shown that *Ichthyobacterium* populations are clonal populations with few variations. Furthermore, if low-cost growth techniques can be created and bacterial quality is steady, it would be possible to develop an effective vaccine. Takano et al. [119] sequenced *Ichthyobacterium seriolicida* isolated from *Seriola quinqueradiata*. This genome data of JBKA-6T sp. is being used in research to create a recombinant vaccine [119].

### 6.3. Bacterial Coldwater Disease

Fish that live in cold, fresh waters with temperatures of 16 °C or lower are susceptible to one of the bacterial diseases known as coldwater disease (CWD). *Flavobacterium psychrophilum* causes bacterial CWD [120] and rainbow trout fry disease [121]. In 1990 and 1993, Japanese researchers documented the presence of bacterial CWD in farmed coho salmon and natural sweetfish, respectively [122,123]. It has significantly harmed the abundance of sweetfish in numerous rivers. Genomics, serotypes, and lethal studies indicated different strains of *F. psychrophilum* in sweetfish and other species [124,125], and this has led to the indication of their immuno-compromised state under infection [126,127].

Antiserum obtained from bacterial CWD-infected fish used for passive immunization helps to battle illness and can be used to develop an efficient vaccine [128]. The use of an oil-based adjuvant, formalin-killed cell vaccine, has also demonstrated a protective effect [129]. This oil-based adjuvant takes at least two months to become completely washed off from the bodies of sweetfish. Since sweetfish are annual and all their organs are edible, this long-term residual adjuvant vaccine is unsuitable [130]. Further developments of a water-soluble adjuvant and its effectiveness with a short residence period have also been demonstrated [131,132,133].

Unfortunately, the adjuvant’s toxic properties prevented the vaccine from being marketed. No permanent impact has been attained despite studies on enteric micro-capsulated oral vaccinations and oral vaccines employing formalin-killed cells from logarithmic bacterial colonies [132]. Researchers have focused on recombinant vaccines of antigenic proteins against *F. psychrophilum* in sweetfish [133]. Although these antigenic proteins are promising candidates in the creation of bacterial CWD vaccines, further research is required to improve the efficacy of these vaccines [134].

### 6.4. Erythrocyte Inclusion Body Syndrome

The etiologic agent of EIBS in *Oncorhynchus kisutch* is porcine orthoreovirus 2 [135]. The illness strikes when the water temperature is below 10 °C, resulting in anemia and widespread deaths. PRV-1 is shown to be linked to PRV-2 [136,137] and PRV-3 [138], which are infections that cause HSMI in *Salmo salar* and resemble HSMI in rainbow trout [139]. Although there have been no serious attempts to culture porcine orthoreovirus 2, a DNA-based genetic approach may help to develop a vaccine. Haatveit et al. [140] studied the PRV-1 DNA vaccine in experimental fish, which showed a moderate protective effect. A recombinant vaccine might work for EIBS due to the correlation between PRV-1 and PRV-2.

### 6.5. Parasitosis

In Japan, farmed marine fishes are plagued by the “skin-parasitic capsalid monogeneans, *Neobenedenia girellae* and *Benedenia seriolae*”. The latter has strong host specificity for *Seriola* species, while the former parasitizes various fish species and exhibits moderate host specificity [141]. Even though praziquantel oral administration and bath treatments with freshwater or hydrogen peroxide solutions successfully control the parasite, they involve significant cost or work [142]. In numerous marine fish species, including the Japanese flounder (*Paralichthys olivaceus*), the ciliate *Miamiensis avidus* causes scuticociliatosis [143]. No effective treatment has made a vaccine compulsory for scuticociliatosis infection in several countries, like Malaysia and Japan [144]. The death rate was reduced after infections with ciliate to *P. olivaceus* immunized with formalin-killed peracetic *M. avidus* [143]. However, a vaccination study with *Philasterides dicentrarchi* or *M. avidus* in Spanish turbot indicated their effective protection when used in vaccine form [145,146]. Therefore, three different *M. avidus* or *P. dicentrarchi* serotypes are used in Malaysia and Japan [144].

Surface antigens implicated in *M. avidus* immobilization have been identified as highly antigenic, short, linear peptide fragments of proteins that could be associated with three serotypes [146]. As a result, it is believed that a combination of different immobilized *M. avidus* serotypes or polypeptides generated from different serotypes are potential antigens for vaccines.

### 6.6. Vaccines Other Than Inactivated Vaccines

Eel, Japanese flounder, and red sea bream aquaculture in Japan suffer commonly from Edwardsiellosis [147]. It was already established that cultured yellowtail and greater amberjack are negatively impacted by infectious diseases brought on by *Mycobacterium* spp. and *N. seriolae* [148,149,150].

Mochizuki et al. [151] specified that the nucleotide of IHNV isolates mutates quickly. It modulates its virulence capacity in rainbow trout farms, as observed in countries such as Japan and Malaysia. Various scientists worldwide are working to develop preventative measures against these intracellular infections. *Edwardsiella*, *Mycobacterium*, *N. seriolae,* and IHNV are infections occurring within a cell. To prevent these intracellular infections, a live-attenuated vaccine or a cytokine adjuvant-based vaccine has been established [113,152]. A DNA vaccine for IHNV infections has been approved against intracellular pathogens of fish in the USA and Canada. The rules for introducing additional vaccinations in fish have not yet been studied. In Japan, all known licensed fish vaccines are inactivated ones. Therefore, it is essential to build an administrative structure that can integrate live-attenuated or DNA-based vaccines into Japanese aquaculture and confirm their safety and efficacy [153]. Major disease prevention could be achieved using complete vaccination protocols in aquaculture. Major vaccination is used in the commercial aquaculture of species like barramundi, rainbow trout, Atlantic salmon, sea bass, sea bream, tilapia, turbot, and yellowtail. Currently, available vaccines for commercial aquaculture are listed in Table 6.

Additionally, experimental vaccinations are used to prevent illnesses, including *Vibrio harveyi* infection in barramundi and *Photobacterium damsela* subsp. damsela infection in salmonids, piscirickettsiosis, and bacterial kidney disease in salmonids, as well as *Flexibacter maritimus* infection in turbot. However, non-licensed vaccines remain utilized in industrial fish farms. Many approved bacterial vaccines are inactivated products, and only a small number of recombinant vaccine technologies have been applied thus far. Typically, intraperitoneal injections of multivalent vaccinations are used to immunize salmonid fish. Although booster vaccination is increasingly used predominantly in the Mediterranean province, immersion vaccination remains the standard immunization method for marine fish species [64,156,157].

### 6.7. Autogenous Vaccines

Japan forbids the use of autogenous vaccinations in aquaculture. Inactivated immunological VMPs made from pathogens and antigens taken from an animal or animals at a particular facility and used to treat that animal or those animals in the same area are known as autogenous vaccines [158]. A publicly funded facility working on a specific disease can produce an autogenous vaccine faster than a commercial vaccine, which can be created and granted a license [159] (Figure 2). Therefore, when there are no reports of effective commercial fish vaccines, these autogenous vaccines prepared from the pathogen strains in a precise fish farm may provide protection. Various diseases and their variations can typically arise when cultivating different fish species. The advancement of generating a fish vaccine may be appropriately aided by introducing autogenous vaccines in nations like Japan, where various fish species are cultured [160,161,162,163]. Therefore, more research on fish vaccines is now needed.

## 7. Plant-Derived Fish Vaccines—A New Perspective in Immunology

Plant molecular farming is an emerging expression system through which recombinant genes are expressed in plant cells. In this technique, foreign proteins are expressed in plants with the purpose of using only the protein rather than the plant [164]. In the late 1980s, attempts were made to express recombinant proteins in plants [165,166]. Initially, plants were used to express pharmaceutically essential proteins. In 2012, a plant-produced enzyme, Glucocerebrosidase, was the first plant-expressed product commercially available for human use [167]. Since then, numerous research has been conducted to produce human and animal recombinant proteins in plants. Three different platforms are used to express the recombinant protein in plants: stable nuclear expression, stable expression of transgenes in chloroplasts, and transient expression of transgenes [168] (Figure 3). Each approach has its benefits and drawbacks, and the choice of platform is mainly determined by the amount of required protein.

Currently, good manufacturing practices and regulatory concerns with plant-made recombinant proteins have also been thoroughly developed. Plant molecular farming may utilize a wide range of plant species. In lettuce, tomato, potato, cabbage, and other edible plants, nuclear and plastid genome engineering has been used to produce a variety of recombinant proteins. It has been suggested that a chloroplast expression system is a promising method of producing oral vaccinations. Vaccines against viruses, bacteria, and parasites have been made using transgenic plants [168].

### 7.1. Advantages of Plant-Derived Fish Vaccines

Plant-derived vaccines have been used for their multi-fold advantages such as the following: (1) the plant expression system is safe, and the vaccines do not have any toxic metabolites and hyperglycosylated proteins; (2) plants have a greater capacity for the biosynthesis of proteins on a large scale and perform complex assembly and folding of proteins; (3) plants are environmentally friendly, cost-effective, and sustainable when compared to existing expression systems; and (4) plant-derived vaccines can be effectively used for oral delivery without the requirement of sophisticated and time-consuming downstream processes.

### 7.2. Prospective Plant-Derived Fish Vaccines

The financial cost is crucial to consider while developing vaccines for the aquaculture industry. The plant expression system for vaccine production provides cost-effectiveness, safety, and efficacy. Plant-derived fish vaccines will provide increased and sustainable fish health in the flourishing global aquaculture industry. Research on plant-derived fish vaccines has been scarce, but the numbers of studies are now growing [169]. In aquaculture, there is much potential for oral vaccinations made from edible plants for fish. Additionally, a plant-generated recombinant vaccine can simultaneously deliver multiple antigenic proteins [170]. Table 7 shows some plant-derived vaccines in fish and other animals. Nevertheless, there is currently an absence of commercially available fish vaccines produced from plants (Figure 3). Therefore, it is necessary to conduct more studies on the development of fish vaccines employing plants as an important source [171].

Oral immunization offers a stress-free, time-saving means of delivery, with fewer labor costs, and minimizes the need for expensive downstream processing, purification, and cold storage while transporting [176]. Using a plant as an expression system, recombinant major capsid protein (rMCP) from iridovirus affecting *Neoscorpis lithophilus* was successfully expressed in rice callus. The rMCP was able to provide immune protection from iridovirus [62]. Likewise, nervous necrosis virus (NNV) coat protein was stably transformed into *N. tabacum* chloroplasts [58]. AcrV and VapA antigens from *Aeromonas salmonicida* affecting salmon were stably expressed in chloroplasts of *Chlamydomonas reinhardtii,* showing elevated immune production during infection [172]. White spot syndrome virus (WSSV) affecting crustaceans causes substantial economic loss, and the VP28 protein from WSSV was successfully expressed in *Chlamydomonas reinhardtii* and *Dunaliella salina* [173,174]. A successful transient expression of Atlantic cod nervous necrosis virus (ACNNV) VLPs in *N. benthamiana* was demonstrated, and an immunization study showed its effective defense against a virus challenge [175]. Plant-based virus-like particles (VLPs) against piscine myocarditis virus (PMCV), causing cardiomyopathy syndrome in wild Atlantic salmon, was transiently expressed in *N. benthamiana*. However, the plant-derived PMCV VLP vaccine induced limited immune protection against PMCV infection [170].

**Figure 4:**
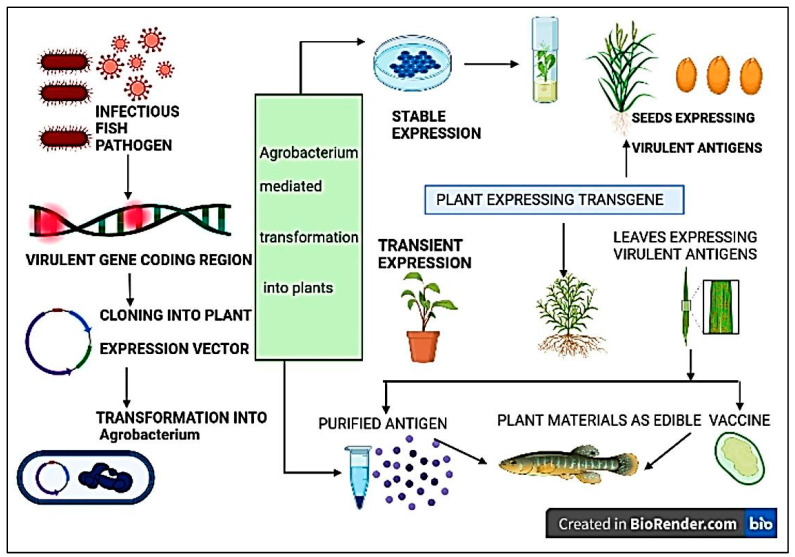
Schematic representation of the steps involved in plant-based fish vaccine development.

To elicit effective immune responses in fish, studies on plant-derived vaccines primarily focus on enhancing the quantity and purity of the produced antigens in the expressing plants (Figure 4). For the maximum amount and quality of antigens, targeting the most appropriate subcellular compartment in plant cells is crucial. Although there are numerous obstacles to overcome in the field of plant-derived vaccine production and application, the potential and appeal of enhanced plant-based immunizations are undeniable. Ultimately, plant biotechnology presents a promising option for developing future aquatic vaccines.

## 8. Updates on Strategies to Develop Fish Vaccines

A vaccine should be safe for a fish, and the mode of administration, if it is the oral mode, should be better and provide long lasting better protection. The main challenge to producing and developing vaccines remains in a sequence of events, from manufacturing to marketing. Another critical challenge is providing a cost-effective and readily available vaccine [176]. A significant restriction is the commercialization of oral vaccines and preservation of antigens. The licensing of vaccines is also not practically applicable for all fish antigens. Autogenous vaccines can be used as an alternative. Brooker et al. [177] studied an autogenous vaccine that was shown to be effective in controlling *Aeromonas salomonicida* when administered via injection in cleaner fish. Adjuvants are limited for mucosal vaccines, and adjuvant usage modulates the immunogenicity of an antigen [11]. Vaccines must be designed according to the type of fish species, production cycle, farming technology, environment, etc. Strategies must be developed based on the genomes of various fish, and the epidemiology with a multi-approach strategy needs to be taken care of for better production (Figure 4) [11].

Kole et al. [178] proposed that a trivalent oral vaccine consisting of attenuated viral hemorrhagic septicemia virus (VHSV), *S. parauberis* serotype I, and antigens of *M. avidus*, encapsulated in a chitosan–PLGA complex, could be an assuring strategy to prevent the outbreak of diseases in olive flounder. In addition, various other vaccination approaches are in their developmental phases, such as algal-enclosed oral vaccines, bacterial biofilm-based antigens, and exosome-derived vaccines [179,180,181]. Partial immunization in fish has led to the utilization of epitope-based vaccines, where one could, with the help of bioinformatics, formulate an antigen with multiple epitopes that would be potentially beneficial in creating effective vaccines [182]. Orally delivered vaccines have the disadvantage of disintegrating in the (acidic) gut of fish. As mentioned earlier, when encapsulated by a microalgal cell wall, these vaccines or recombinant proteins are protected from such harsh environments and are delivered to the system very easily via cell wall degradation [179].

**Figure 5:**
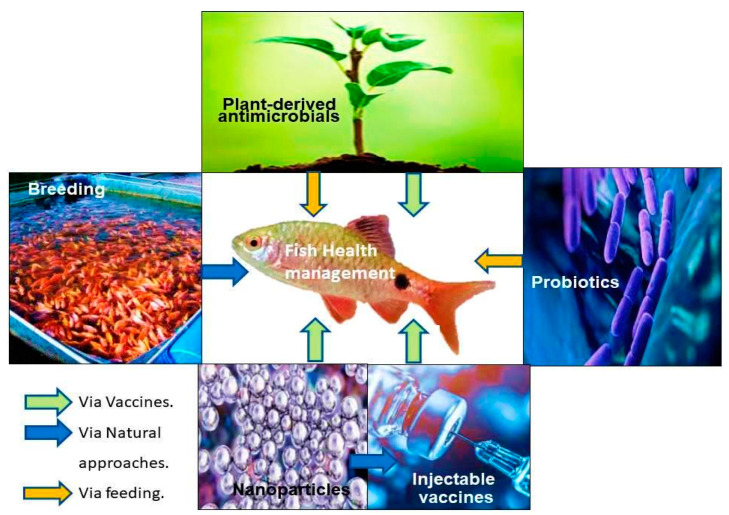
Control and prevention strategies for infectious diseases in fish [25,183]. Different approaches are used to maintain fish health, such as nano-based drugs/vaccines, injectable vaccines, probiotics, plant-based medicines, or edible vaccines.

Control and prevention strategies must be designed to appropriately treat infectious diseases in fish. Xu et al. [183] highlighted the necessity of employing multi-strategy approaches for fish production, emphasizing disease prevention, climate control, environmental management, and planned governance to meet the anticipated demand by 2050 for the national and global market distribution of fish products. This can be achieved with the geographical expansion of fish species, fishing capacity building, natural variability, and the employment of judicious culture techniques with newer science, policies, and interventions [183]. As per Xu et al.’s [183] report, although applicable measures have been identified, fewer vaccines are available for fish disease prevention and management. General prevention strategies are highlighted in Figure 5. Another major challenge in developing novel techniques is antibiotic resistance by microorganisms in fish.

The world’s need for fish is increasing every day, and the estimated demand for fish by 2050 is presented in Table 8. Therefore, loss due to infectious diseases in aquaculture must be controlled. Various vaccines proven to have a positive impact on reducing fish mortality have been outlined in this review. However, the most effective vaccines are necessary to address the increased demand for aquaculture, especially the need to manage large-scale fish farming.

Global capture-based aquaculture production has been recorded throughout various periods in different nations, revealing a slight decline in production in 2020 in the inland aquaculture system. Moreover, food security and sustainability concerns will increase as the world population approaches 9 billion by 2050 (Table 8) [184]. Therefore, economic expansion, job growth, nutritional profiles, and gender empowerment are a few variables that have contributed to the increased demand for aquaculture. The market expanded approximately 20-fold in 2020, compared to production in 1976. Billions of people depend on fishing and its allied sectors to meet their financial and health needs. The increase in fish consumption is primarily driven by technological developments in processing and distribution, directly affecting the increased demand for fish and fish products. Good management practices are crucial to reduce stress and minimize the prevalence of diseases (Figure 4 and Figure 5). Marine ecosystems are constantly bombarded with challenges that seriously threaten their sustainability. Several studies assert that climate change is the major player among all other factors [12]. Currently, there seem to be no climate-change measures that have been added to fish aquaculture management policies [185]. A shift in the regulatory guidelines of aquaculture has only occurred recently, with governments opting for a more ecosystem-based approach to aquaculture management (EAF). In simple terms, EAF considers all the species of a particular marine ecosystem rather than focusing on one species in isolation. Other components such as climate change, co-interactions with other species, and pollution also come into the picture (“Understanding Ecosystem-Based Fisheries Management,” National Oceanic and Atmospheric Administration). Understanding the role of each factor helps maximize the benefits of fish aquaculture and other marine ecosystems by avoiding over-exploitation of the available resources. It is vital to ensure that aquaculture is managed appropriately to attain the goals of food security, employment, and nutritional benefits. Apart from EAF, other existing strategies for managing aquaculture include rights-based management and management for resilience [186]. Therefore, fish vaccination is one of the significant tools for the management of aquaculture health. Hence, it is crucial to redirect attention away from traditional vaccines and instead prioritize novel fish immunization techniques. Vaccine development should employ advanced technologies like “Omics based” [187,188] and nano-carrier-based adjuvants [189,190] and environmentally friendly vaccines like plant-based vaccines [76,190], which are efficient and cost-effective, require small doses, and do not require the use of antibiotics. Such research on vaccine development is necessary in light of the increasing importance of aquaculture globally in recent years [16,191,192].

## 9. Conclusions

The demand for aquaculture products is growing faster than expected internationally. With the ever-growing demand for fish in world markets, their management in aquaculture by preventive measures against contagious diseases is a challenging area. Therefore, modern technology is presently employed in developing unique and alternative fish vaccines—for example, plant-derived vaccines—which help develop immune responses beneficial to fish health. The industry requires reliable aquaculture vaccines for economic success. The inefficient steps of conventional vaccine-developing methods must be modified to fight against emerging disease concerns. Research protocols for producing fish vaccines that employ newer technologies may be expensive but are essential. It will be crucial to leverage the abundance of biotechnology that is now available to address emerging disease concerns due to the continual demand for novel vaccines induced by the global development of aquaculture. In particular, vaccination in broodstocks is essential for fish seed production. The development of fish vaccines is known to use either whole-killed pathogens; subunits of a protein, peptide, or recombinant protein; DNA vaccines; or live-attenuated vaccines via different modes of administration. Further research is anticipated to explore strategies regarding the mode of administration and use of adjuvants.

## Figures and Tables

**Table 1:** Different infectious agents, diseases they cause, and commercial vaccines against them in animals.

S. No.	Infectious Agent/Etiological Agent	Diseases	Animals Infected	Vaccines Used to Prevent the Disease (Either Used in Humans or Animals)
1.	*Avian Influenzae* virus	Avian Influenza	Poultry: chickens, ducks, turkeys, geese	Afluria Quadrivalent, Fluarix Quadrivalent, FluLaval Quadrivalent, and Fluzone Quadrivalent
2.	*Herpes* virus, Gallid alphaherpesvirus 2	Marek’s disease (fowl paralysis)	Poultry: chickens	HSV vaccine candidate (mRNA-1608), herpes zoster
3.	*Salmonella* sp.	Salmenollosis	Aquatic animals: fishes; tortoises, birds, cattle, pigs, horses	Ty21a, Vivotif (Typhoid Vaccine Live Oral Ty21a), Typbar TCV^®^, Typhim Vi, Vivotif
4.	*Rabies lyssavirus*	Rabies	Dogs	HDCV or PCEC, human rabies immune globulin (HRIG)
5.	*Bacillus* *anthracis*	Anthrax	Cattle, sheep, goats, camels	Anthrax Vaccine Adsorbed (AVA) or BioThraxTM
6.	*Brucella* bacteria	Brucellosis	Cattle, sheep, goats, swine, equines	Live-attenuated *Brucella abortus* strain 19 (S19 vaccine), *Brucella abortus* S19
7.	*Listeria* *monocytogenes*	Listeriosis	Birds, crustaceans	Under clinical trials
8.	SARS-CoV	Severe acute respiratory syndrome	Bats, birds, cattle	COMIRNATY^®^, COMIRNATY^®^ Original/Omicron BA.1, COMIRNATY^®^ Original/Omicron BA.4-5, VAXZEVRIA, COVISHIELD™, COVID-19 Vaccine, SPIKEVAX, Inactivated COVID-19 Vaccine (Vero Cell), CoronaVac, COVAXIN^®^, COVOVAX™, NUVAXOVID™, CONVIDECIA
9.	Nipah virus (NiV)	Nipah	Bats, pigs, horses, goats, sheep	Nipah Virus Vaccine (PHV02)
10.	Monkey pox virus	Monkey pox	Rope squirrels, dormice, non-human primates, etc.	ACAM2000^®^, JYNNEOS™ (Imvamune or Imvanex or MVA-BN)
11.	*Clostridium* *botulinum*	Botulism	Fishes, mainly trout	*Fabrizio Anniballi*, *Alfonsina Fiore*, *Charlotta Löfström*, *Viveca Båverud*.
12.	*Mycobacterium* sp.	Mycobacterial infections	All fish	*Bacille* Calmette-Guérin (BCG)
13.	*E. coli*	Avian bacterial infections (AVECs)	All avian sp.	Poulvac *E. coli*

A list of different causatives and infectious or etiological agents is given in this Table. Susceptible animals indicate that the adaptation of vaccination is recommended in animals; data adapted from The World Health Organization [1].

**Table 2:** Communicable diseases, associated pathogens, symptoms, and treatments in different fish.

S. No.	Disease	Pathogen	Symptoms	Treatment	References
1.	ColumnarisDisease	*Flavobacterium columnare*	Lesions in skin, fin erosion, necrosis in gills	Amphenicol, Nifurpirinol, Nifurprazine, Oxolinic acid	[35,36,37,38,39,40,41]
2.	Epizootic ulcerative syndrome (EUS), or “red spot disease”	*Aphanomyces* *invadens*	Red lesions (sores) or deep ulcers	No effective treatment but can be treated with different parts of *Azadirachta indica*	[42]
3.	Spring viremia of carp	Rhabdovirus, spring viremia of carp virus	Destruction of kidney, spleen and liver tissues	DNA vaccination may be protectable in fish	[43,44,45]
4.	Lymphocystis	*Lymphocysti* virus or *Lymphocystis* disease virus	Pebble- or wart-like nodules mostly on the fins, skin, gills, etc.	No effective treatment	[46,47]
5.	Carp pox	Cyprinid herpesvirus-1 (CyHV-1)	Milky skin lesions, thickening of fins	No effective treatment	[48,49,50,51,52,53,54,55,56,57,58,59,60]

**Table 3:** Commercial names of a few fish vaccines and their mode of administration.

Species	Disease	Organism	Name of the Vaccine	Mode of Administration	Type of Vaccine	Reference
Salmon	Infectious hematopoietic necrosis	Infectious hematopoietic necrosis virus	APEX-IHN^®^	IM	IHNV plasmid vaccine	[57]
	Enteric red mouth disease, yersiniosis	Yersinia *ruckeri* serotype O1b	Alpha ERM Salar	IP	Inactivated bacterial vaccine	[58]
			Aquavac YER knows	IP	Inactivated bacterial vaccine	[59]
	Pancreatic disease	Salmon pancreas disease virus	ALPHA JECT micro 1 PD	IP	Inactivated viral vaccine	[58]
		Salmonid alphavirus	Clynav	IM.	DNA plasmid	[60]
		Salmon alphaviruses (SAV)	PD Norvax^®^ Compact PD	IP	Inactivated viral vaccine	[59]
	Infectious salmon anemia	Infectious salmon anemia virus (ISAV)	ALPHA JECT^®^ micro 1 ISA	IP	Inactivated viral vaccine	[58]
Tilapia	Streptococcosis	*S. agalactiae* serotype lb	AQUAVAC^®^ Strep Sa	IP	Inactivated viral vaccine	[61]
		*S. agalactiae* serotype Ia and serotype III	AQUAVAC^®^ Strep Sa1	IP	Inactivated viral vaccine	[61]
Tilapia, seabass		*S. iniae*	AQUAVAC^®^ Strep Si	Dip immersion/IP	Inactivated viral vaccine	[61]
		*Streptococcus agalactiae* Ib	ALPHA JECT^®^ micro 1 Tila	IP	Inactivated viral vaccine	[58]
Koi	Koi herpes virus disease	Koi herpes virus (KHV)	KV-3	Immersion/Injection	Attenuated viral vaccine (not used because of its safety issues)	[61]
Sea bass	Viral nervous necrosis	Nodavirus	ALPHA JECT micro^®^ 1 Noda	IP	Inactivated viral vaccine	[58]
	*Aeromonas veronii* infection	*Aeromonas veronii*	Autogenous *Aeromonas veronii* vaccine	IP	Inactivated bacterial culture	[58]
	Vibriosis	*Listonella anguillarum*	ALPHA DIP^®^ Vib	Dip vaccine	Inactivated bacterial vaccine	[58]
Asian seabass	Epizootic hematopoietic necrosis	Iridovirus	AQUAVAC^®^ IridoV	IP	Inactivated viral vaccine	[61]
Seabass, rainbow trout	Infectious pancreatic necrosis	Infectious pancreatic necrosis virus (IPNV)	AquaVac^®^ IPN Oral	Oral	Inactivated viral vaccine	[59]
			Alpha Jects^®^ 1000	IP	Inactivated viral vaccine	[58]
Pangasius	Enteric septicemia disease, motile aeromonad septicemia	*Aeromonas hydrophila* and *Edwardsiella icataluri*	ALPHA JECT^®^ Panga 2	IP	Inactivated bacterial vaccine	[58]
Gray mullet (*Mugil cephalus*), Nile tilapia	Lactococcosis	*Lactococcus garvieae*	Ichtiovac-Lg	IP	Inactivated vaccine	

**Table 4:** List of vaccines proved to be preventing infections in fish.

Types of Vaccine	Name of Vaccine	Fish	Infection	Remark
Inactivated or heat-killed whole-cell vaccine	Apha Ject^®^ 1000, Norway	Salmon	Infectious pancreatic necrosis virus	Monovalent
	Inactivated SVCV	Carp	Spring viremia of carp virus	SVCV emulsified in oil
	Formalin-inactivated IHNV	Rainbow trout	Infectious hematopoietic necrosis virus	
	Killed VHSV	Rainbow trout	Viral hemorrhagic septicemia virus	--
Attenuated vaccines	Attenuated KHV Israel	Carp	Koi herpes virus	
	Attenuated *Flavobacterium columnare*	All freshwater finfish	*Flavobacterium columnare*	--
	Septicemia due to enterococci	Catfish	*Edwardsiella ictaluri*	--
	Kidney infection in fish due to bacteria	Pacific salmon and Atlantic salmon	*Renibacterium salmoninarum*	--
Recombinant vaccines	Recombinant G protein	Carp	SVCV	--
	CIBA-Nodavac-R	All types of fishes infected with nervous necrosis virus (NNV)	Red-spotted grouper NNV	First vaccine indigenously developed in India
Synthetic peptide vaccines	Subunit vaccine IPNV aquabirnavirus	Rainbow trout and Atlantic salmon	Infectious pancreatic necrosis viruses	Target: VP2, VP3 and Capsid proteins
DNA vaccines	Apex IHN, Canada	Salmon	Viruses having G antigen	
	DNA	Salmonids	Infectious hematopoietic necrosis rhabdovirus	Target: G glycoprotein
Mucosal vaccines	MicroMatrix™ delivery system (*Piscirickettsia salmonis*, ISAV and IPNV, Centrovet)	Atlantic salmon	*Y*. *ruckeri V*. *anguillarum*, *P. salmonis* and IPNV or other similar mucosal infection.	Pathogen killed by heat or inactivated by formalin
Plant-based edible vaccines	Under development in plant *Nicotiana benthamiana*	Salmonids	PMCV and cardiomyopathy syndrome	--
Nanoparticle-based vaccines	Chitosan-NPs based vaccine formulation NPrgpG, pICrgpG, CSrgpG, NpiV	Zebrafish	Viral hemorrhagic septicemia virus.	Under experimental trials
	OCMCS-hyaluronic acid, OCMCS/aerA-NPs, OCMCS-HA/aerA-NPs	European carp	*Aeromonas hydrophila*	Under experimental trials
Monovalent and polyvalent vaccines	ME-VAC Aqua Strept	Nile tilapia fish, Nile tilapia	*Streptococcus* infections	Effective against bacterial strains, *Streptococcoci*, *Enterococcoi*, and *Lactococci*

Table 4 shows the “available” list of fish vaccines. Data are collected from Mondal and Thomas [65] and Dhar et al. [60].

**Table 5:** Some examples of infectious diseases affecting fish that cause economic losses in the aquaculture sector.

Sr. No.	Causative Agent	Diseases	Type of Fish Infected	Loss in Production/Economic Loss (%)	References
1.	*Aeromonas* bacteria	Aeromonas infections	Carps, freshwater fish	80–100%	[93]
2.	*Pseudomonas* sp.	Strawberry disease	Carps, rainbow trout, tench	50%	[94]
3.	*Shewanella* *putrefaciens*	Shewanellosis	Carps, rainbow trout, zebra fish	-	[93,94]
4.	*Mycobacterium* sp.	Mycobacteriosis	All freshwater and marine fish	50%	[95]
5.	*Flavobacterium* *flavobacter*	Bacterial gill diseases	All fish	60–70%	[96]
6.	*Birnavirus*	Necrosis	Freshwater fish like salmonids	50%	[25]
7.	*Retrovirus*	Anemia	Walley pike	50%	[97]
8.	Ranavirus	Anemia	Carp	50%	[98]
9.	Megalocytivirus	Anemia	Carp and other freshwater fish	60–70%	[98]

Due to the severity and varied symptoms of diseases, the total production and diversity of fish are hugely affected, especially in India. Disease outbreaks are mainly due to malpractices in aquaculture. The mentioned causative agents have the ability to cause diseases in a variety of animals and birds as well.

**Table 6:** Major vaccinations used in commercial aquaculture systems.

S. No.	Species	Vaccination against	Reference
1	Cold-water vibriosis, Classical vibriosis,	*Listonella anguillarum, Vibrio ordalii*	[154]
2	Furunculosis	*Aeromonas salmonicida* subspecies *achromogenes*	[155]
3	Vibriosis	*Vibrio salmonicida* *Vibrio anguillarum*	[155]
4	Yersiniosis	*Yersinia ruckeri*	[154]
5	Pasteurellosis	*Photobacterium damselae* subspecie *piscicida*	[154]
6	Edwardsiellosis	*Edwardsiella ictaluri*	[154]
7	Winter ulcer	*Moritella viscosa*	[154]
8	*Streptococcosis/Lactococcosis*	*Streptococcus iniae, Lactococcus garviae*	[154]

**Table 7:** List of plant-derived vaccines in fish and other animals under development.

S. No	Protein Expressed	Expression System	Treated Animals	Reference
1.	Recombinant major capsid protein (rMCP) of iridovirus	Rice callus	*Neoscorpis lithophilus*	[160]
2.	Nervous necrosis virus (NNV) coat protein	Tobacco chloroplast	Grouper fish	[58]
3.	AcrV and VapA antigens from *Aeromonas salmonicida*	Chloroplasts of *Chlamydomonas reinhardtii*	Salmon	[172]
4.	VP28 from white spot syndrome virus	*Chlamydomonas reinhardtii*	*Penaeus monodon*	[173]
5.	VP28 from white spot syndrome virus	*Dunaliella salina*	Crayfish	[174]
6.	Virus-like-particle from Atlantic cod nervous necrosis virus (ACNNV)	*Nicotiana benthamiana*	Salmonids	[175]
7.	ORF1 from cardiomyopathy syndrome (PMCV)	*Nicotiana benthamiana*	Salmonids	[170]

**Table 8:** Prediction for fish demand for human consumption by 2050 in the top ten fish-producing countries.

World Position Number	Country	Total Consumption (Million Tons)
1	China	99,875
2	India	24,601
3	United States	10,423
4	Mexico	6061
5	Brazil	5460
6	Nigeria	5359
7	France	3494
8	Spain	2529
9	Peru	23,331
10	Ghana	9121

## Data Availability

All data generated or analyzed during this study are included in this published article.
